# Test for Olive Oil

**Published:** 1863-11

**Authors:** 


Test for Olive Oil.—It is a well-known fact that olive oil sold in America and elsewhere is very seldom pure, but mostly adulterated with other cheap vegetable oils. M. Hauchecorne, chemist, of Yvetot, France, has, however, just discovered an easy method of detecting such frauds. M. Hauchecorne's test is oxygenized oil, which may be had at any chemist's, and is a colorless liquid. The method of using it consists in pouring a portion of the oil to be tested into a graduated tube, by which the volume of the liquid is ascertained at a glance. To three volumes of olive oil one volume of oxygenized oil is added, and the whole well shaken. After a few seconds the mixture will become green, if the oil is pure olive. No other oil will do so. Poppy oil will assume a rose color with the test; oil of sesamum will turn bright red; oil of arachis turns yellowish gray; the beech-nut oil takes the color of ochre. These are the oils most commonly used to adulterate olive oil. When the latter is pure, the green tint appears instantaneously; but if adulterated, the liquid must stand three or four minutes before the color, whatever it may be, appears. Even the quality of the olive oil, when pure, may be tested in this way. Thus, an apple-green color shows that the olives employed were too ripe. A light green denotes oil obtained from a mixture of olives of different qualities, but none of them spoilt. The olive oils of Nice, Port Maurice and Genoa belong to the former sort; those of Aix and Grasse to the latter.—*Medical and Surgical Reporter.*

